# Impact of in ovo administration of xylo- and mannooligosaccharides on broiler chicken gut health

**DOI:** 10.1016/j.psj.2024.104261

**Published:** 2024-08-24

**Authors:** Aleksandra Bełdowska, Maria Siwek, Jakub Biesek, Marcin Barszcz, Anna Tuśnio, Kamil Gawin, Aleksandra Dunisławska

**Affiliations:** ⁎ Department of Animal Biotechnology and Genetics, Faculty of Animal Breeding and Biology, Bydgoszcz University of Science and Technology, Mazowiecka 28, 85-084 Bydgoszcz, Poland; † Department of Animal Breeding and Nutrition, Faculty of Animal Breeding and Biology, Bydgoszcz University of Science and Technology, Mazowiecka 28, 85-084 Bydgoszcz, Poland; ‡ Department of Animal Nutrition, The Kielanowski Institute of Animal Physiology and Nutrition, Polish Academy of Sciences, Instytucka 3, 05-110 Jabłonna, Poland

**Keywords:** microbiota, prebiotic

## Abstract

The intestinal mucosa creates a connection between the gut microbiota and the host. This study aimed to modify the gut microbiota of broiler chickens by in ovo stimulation with xylo-oligosaccharide (**XOS**) and manno-oligosaccharide (**MOS**) prebiotics and to determine the changes occurring in specific gut segments. Three hundred incubated eggs of Ross 308 broiler chickens on the 12th d of incubation were injected with: saline (control), xylotriose (**XOS3**), xylotetrose (**XOS4**), mannotriose (**MOS3**) or mannotetrose (**MOS4**). Tissue and digesta samples were collected post-mortem from 8 randomly selected individuals from each group, on d 42 after hatching. Gene expression analysis in the cecum and ileum was performed by RT-qPCR for a panel of genes: innate immune response genes (*IL-2, IL-4, IL-6, IL-8, IL-10, IL-12, IL-17, IL-1β, IFNγ, IFNβ*), nutrient sensing and nutrient transport genes (*FFAR2, FFAR4, GLUT1, GLUT2, GLUT5*), host defence peptides (*AvBD1, CATHL2*), and barrier function genes (*MUC6, CLDN1, TJAP*). The relative abundance of bacteria was determined by qPCR for individual bacteria (*Akkermansia muciniphilla, Bifidobacterium* spp., *Clostridium difficile, Escherichia coli, Faecalibacterium prausnitzii,* and *Lactobacill*us spp.). Stimulation with prebiotics caused changes in the abundance of bacteria especially *Lactobacillus* spp. and *Bifidobacterium* spp. in the cecum. The abundance of both genera increased in each study group compared to the control group. The highest abundance of *Bifidobacterium* spp. in the ileum was found in the MOS3 group compared to the control group. There were changes in the XOS4 and MOS3 groups in the expression of: *FFAR4, GLUT1, AvBD1, CATHL2, IL-2, IL-12*, and *IL-17* in the caecum. In conclusion, in ovo administration of prebiotics increased intestinal colonization by bacteria. The prebiotics influenced gene expression levels via changes in the gut microbiota.

## INTRODUCTION

The intestinal mucosa is the first line of defense, protecting the epithelial surface from pathogens and mechanical damage during digestion (Duangnumsawang et al., 2021). Mucus is responsible for stimulating colonization by commensal bacteria and providing an optimal environment for digestion and simplifying nutrient transport. The intestinal mucosa is densely colonized by microorganisms capable of metabolic activity (Forder et al., 2007). The intestinal mucosa should act as a barrier, trapping and immobilizing pathogens while at the same time allowing nutrients to penetrate the epithelial surface (Duangnumsawang et al., 2021). The intestinal mucosa includes the lamina propria, epithelium, and smooth muscle. The epithelium of the small intestine is composed mainly of cylindrical epithelial cells (enterocytes) alternating with goblet cells (Slawinska et al., 2019). The sensory system of immune and intestinal cells recognizes bacteria and their metabolites. This leads to the activation of the host's innate immune response, which involves secretion of cytokines: interleukin (***IL****)-1β, IL-4, IL-13*, and *IL-20*. Lymphoid tissue, in turn, forms the intestinal immune barrier. Innate gut barrier mechanisms include cytokines, mucins, and host defence peptides (**HDPs**) (Slawinska et al., 2019; Duangnumsawang et al., 2021). The intestinal microbiota plays an important role in maintaining intestinal health and influencing the overall performance of chickens. Under undisturbed homeostasis of the host body, intestinal bacteria mainly colonize the outer layer of mucus. They break down mucin proteins and glycans, using them as a potential energy source. Under undisturbed conditions, the inner layer is an impenetrable barrier to bacteria (Khan et al., 2020; Josenhans et al., 2020).

Environmental factors largely influence the composition of the microbiome. Metabolites produced by the microbiome, which include short-chain fatty acids (**SCFA**), are involved in host-microbiome communication and are responsible for maintaining barrier function and immune homeostasis. To optimize the gut microbiota, stimulation with bioactive substances, including prebiotics, are being used. Prebiotics are mostly oligosaccharides that are not digested by host enzymes. They make their way to the downstream (lower) parts of the intestines, where they promote the growth and proliferation of microorganisms. Feeding prebiotics to poultry strengthens the intestinal microbiota by improving host performance and activating resistance to colonization of intestinal pathogens such as *Salmonella* and *Campylobacter*. The most common prebiotics include galacto-oligosaccharides, manno-oligosaccharides, fructo-oligosaccharides, and xylo-oligosaccharides (De Maesschalck et al., 2015; Khan et al., 2020). The aim of this study was to modify the host gut microbiota by in ovo stimulation on d 12 of egg incubation with xylo-oligosaccharide and manno-oligosaccharide prebiotics, and to determine the changes occurring in intestines (ileum and caecum) in broiler chickens after stimulation.

## MATERIALS AND METHODS

### Experimental Setup

Hatching eggs from the parent flock of Ross 308 broiler hens were purchased from a commercial hatchery, the total number of eggs was 700. On d 7, the eggs were candled (ovoscope, Fermo, Piotrów, Poland), and 300 fertilized eggs were chosen for the experiment. On d 12 of incubation, the eggs were randomly divided into 5 groups (60 eggs in each group) and injected into the air chamber with 0.2 mL of saline (0.2 mmol/L) (control) or with one of the prebiotic solutions: xylotriose (**XOS3**), xylotetrose (**XOS4**), mannotriose (**MOS3**), or mannotetrose (**MOS4**). Oligosaccharides were administered at 0.5 mg/0.2 mL NaCl solution. Washable, nontoxic white glue “slime elmers” was applied to the resulting holes. Incubation was performed in a single-stage incubator and hatcher (Jarson, Gostyń, Poland). The eggs were incubated for 21 d. From 1 to 18 d, the eggs were kept in the incubator, while from 19 to 21 d in the hatcher. The incubator was set to 37.7°C, 55/60% humidity, and 50/60% ventilation (inlet open). The temperature in the hatcher was 37.5°C, humidity 70%, and ventilation 80%. The incubator was opened daily for control activities, verification of the microclimate, and dynamic short-term cooling due to the technical solution in the used laboratory incubator. In the incubator, the eggs were placed on trays (6 trays – repetitions with 10 eggs per each group) with automatic rotation by 45° every hour. On d 7 of embryonic development, the candling of eggs was performed to eliminate unfertilized eggs or eggs with early dead embryos. Similarly, the eggs were checked on the day of the transfer to the hatcher (d 18). The hatching data are shown in Table 1. After hatching, divided groups of birds were placed in pens on chopped wheat-straw bedding. Animals received feed and water ad libitum*.* Starter, grower, and finisher feeds were purchased from a feed factory. Their composition complied with the feeding standards for broiler chickens according to the nutritional recommendations. The protein content was 22, 20.5, and 19% in starter, grower, and finisher diets, respectively. The metabolizable energy was, on average, 12.50 MJ/kg. The feed contained all the necessary additives for broiler chickens, including vitamins and mineral ingredients. The feed composition was declared by the manufacturer.

**Table 1:** Hatching parameters of broiler chickens stimulated in ovo by different prebiotics.

Item	Group^1^					SEM	*P*-value
	Control	XOS3	XOS4	MOS3	MOS4		
Eggs in total (sum) on d 12	60.00	60.00	60.00	60.00	60.00	0.000	-
Eggs on d 18 (sum)	60.00^a^	60.00^a^	60.00^a^	58.00^b^	60.00^a^	0.091	0.034
Hatched chicks (sum)	57.00	54.00	56.00	54.00	53.00	0.316	0.724
Crippled and weak chicks (sum)	1.00	0.00	0.00	0.00	0.00	0.067	0.452
Unhatched eggs (sum)	3.00	6.00	4.00	4.00	7.00	0.321	0.737
% of chicks hatched from eggs in total	95.00	90.00	93.33	90.00	88.33	1.579	0.724
% of chicks hatched from eggs placed in the hatcher (on d 18)	95.00	90.00	93.33	93.16	88.33	1.607	0.744
% of chicks crippled and weak	1.67	0.00	0.00	0.00	0.00	0.333	0.452
% of eggs not hatched from eggs in total	5.00	10.00	6.67	6.67	11.67	1.604	0.737
% of eggs not hatched from eggs placed in the hatcher (on d 18)	5.00	10.00	6.67	6.84	11.67	1.607	0.744

a,b the mean values marked with different letters in the row differ statistically significantly at *P* < 0.05, SEM, standard error of the mean.

1 XOS3 – xylotriose, XOS4 – xylotetrose, MOS3 – mannotriose, MOS4 – mannotetrose.

### Growth Performance

The rearing lasted 42 d. In each group, 48 broiler chickens were divided into 4 replications (pens) and kept at a stocking density not exceeding 33 kg of livestock per 1 m^2^ of the surface. Environmental conditions were provided for broiler chickens as described by Biesek et al. (2022). A starter diet was used from d 1 to d 14, a grower from d 15 to d 35, and a finisher from d 36 to d 42. The chickens were weighed (**BW**) on d 1, 14, 35, and 42, and feed intake (**FI**) was recorded. Body weight gain (**BWG**) and feed conversion ratio (**FCR**) were calculated based on the data obtained. Viability was also calculated for each group. On d 42, eight randomly selected individuals from each group were sacrificed to collect intestinal mucosa and digesta samples from ileum and cecum.

The experiment was conducted following the applicable regulations in Poland. The slaughter of the birds was carried out under the applicable regulations on the handling of animals during slaughter, including humane treatment. According to directive no. 2010/63/EU of 22 September 2010 on the protection of animals used for scientific purposes, the consent of the Ethics Committee was not required. According to Act of January 15, 2015 on the protection of animals used for scientific or educational purposes (item 266, Journal of Laws of the Republic of Poland) slaughtering to collect tissues and organs from animals, is not a procedure. The chickens were stunned using percussive blows to the head (firm and accurate blows to the head provoking severe damage to the brain). It was done following applicable acts: Council Regulation (**EC**) No 1099/2009 of 24 September 2009 on the protection of animals at the time of killing (mechanical methods) and Directive no. 2010/63/EU of 22 September 2010 on the protection of animals used for scientific purposes (methods of animal killing). Decapitation was performed by cutting off the head between the occipital condyle and the first cervical vertebra. There was rapid bleeding of the carcass.

### Sample Collection

Intestinal mucosa scraped off the epithelium from cecum and ileum for gene expression was collected in a stabilizing buffer (fixRNA, EURx, Gdansk, Poland). Digesta samples for analyses of microbiota activity indices were taken from the distal part of the ileum and ceca and immediately frozen in dry ice. Mucosa and intestinal contents for isolation of bacterial DNA were collected and stored at -20°C until further processing.

### Measurement of Digesta pH and Short-Chain Fatty Acid Analysis

Ileal and cecal digesta pH was measured using a SevenMulti pH-meter (Mettler-Toledo, Warsaw, Poland) after mixing with ultra-pure water in a 1:2 ratio (w/v). Then, pH of the sample was adjusted to 8.2 by adding 1 M NaOH to convert SCFA to their sodium salts. After centrifugation (10 min, 1,800 *g*, room temperature), the supernatants were stored at -20°C until further analysis. The concentration of SCFA was analyzed as described earlier by Barszcz et al. (2011) using the HP 5890 Series II gas chromatograph (Hewlett-Packard, Waldbronn, Germany) with isocaproic acid as the internal standard.

### Ammonia Analysis

Ammonia concentration in the cecal content was measured spectrophotometrically according to Taciak et al. (2015). The absorbance of the color complex formed during the reaction of ammonium ion with Nessler's reagent was measured at 425 nm using a Maxmat PL biochemical analyzer (Erba Diagnostics France SARL, Montpellier, France). The concentration of ammonia was calculated from a standard curve prepared using NH_4_Cl solution.

### Analyses of β-Glucuronidase Activity in Intestinal Digesta

Digesta samples (ca. 0.5 g) were homogenized for 30 s at 18,000 rpm with 2.5 mL of ice-cold potassium phosphate buffer (pH 6.8 at 37°C) with 1% bovine serum albumin. The samples were sonicated and centrifuged (10,000 *g*, 20 min, 4°C). Supernatants were stored at -40°C for further analyses. The activity of bacterial β-glucuronidase was determined spectrophotometrically according to the method described previously by Barszcz et al. (2011), using phenolphthalein β-D-glucuronide as a substrate. The absorbance was measured using a Unicam UV 300 spectrophotometer set at 540 nm.

### Relative Abundance of Bacteria

Total bacterial/stool DNA was isolated from approximately 120 mg of intestinal content of ileum and cecum, which were lysed and purified using the GeneMATRIX Stool DNA Purification Kit (EURx, Gdansk, Poland) according to the manufacturer's instructions. The storage temperature of the DNA samples was -20°C. The extracted DNA was subjected to quantitative and qualitative evaluation by spectrophotometric method using NanoDrop2000 (Thermo Scientific Nanodrop Products, Wilmington, NC). The relative abundances of *Akkermansia muciniphilla, Bifidobacterium* spp., *Clostridium difficile, Escherichia coli, Faecalibacterium prausnitzii,* and *Lactobacillus* spp. in intestinal content were determined using quantitative PCR (**qPCR**) carried out on a LightCycler 480 II System (Roche-Diagnostics, Basel, Switzerland). The qPCR reactions mixture contained SG onTaq qPCR Master Mix (2x) (EURx, Gdansk, Poland), 1 μM of each primer specific to 16S rRNA (synthesized by Sigma-Aldrich, Schnelldorf, Germany) and 20 ng of bacterial DNA template. The thermal profile of the qPCR reaction was carried out as follows: initial denaturation at 95°C for 15 min, followed by 40 cycles of amplification consisting of denaturation at 94°C for 15 s, annealing at 60°C for 30 s, and elongation at 72°C for 30 s. The fluorescence was measured at the end of each extension step. PCR efficiency for each pair of bacterial primers was calculated in the LightCycler 480 II software from a standard curve prepared for 5 dilutions (1x, 0.5x, 0.25x, 0.125x, and 0.0625x) of pooled bacterial DNA template. The relative abundances of the bacteria were calculated as follows:

Relative Abundances [%] = (E universal)^Ct universal^/ (E target)^Ct target^, (Christensen et al., 2014)

where E universal is the efficiency of qPCR with primers for all bacteria, Ct universal is the Ct values for reaction with primers for all bacteria, E target is the efficiency of qPCR with primers specific for target bacteria, Ct target is the Ct values for reaction with primers for target bacteria (*Akkermansia muciniphilla, Bifidobacterium* spp., *Clostridium difficile, Escherichia coli, Faecalibacterium prausnitzii* and *Lactobacillus* spp. (Table 2)).

**Table 2:** Bacterial primer sequences used in qPCR reaction (F- Forward primer; R-Reverse primer).

Bacteria	Primer sequences (Forward/Revers)	Reference
*Universal bacteria*	F: ACTCCTACGGGAGGCAGCAGT R: GTATTACCGCGGCTGCTGGCAC	(Christensen et al., 2014)
*Akkermansia muciniphila*	F: CAGCACGTGAAGGTGGGGAC R: CCTTGCGGTTGGCTTCAGAT	(Candela et al., 2012)
*Bifidobacterium* spp.	F: GCGTGCTTAACACATGCAAGTC R: CACCCGTTTCCAGGAGCTATT	(Christensen et al., 2014)
*Clostridium difficile*	F: TTGAGCGATTTACTTCGGTAAAGA R:TGTACTGGCTCACCTTTGATATTCA	(Penders et al., 2005)
*Escherichia coli*	F: CATGCCGCGTGTATGAAGAA R: CGGGTAACGTCAATGAGCAAA	(Huijsdens et al., 2002)
*Faecalibacterium prausnitzii*	F: ACCATGAGAGCCGGGGGG R: GGTTACCTTGTTACGACTT	(Lund et al., 2010)
*Lactobacillus* spp.	F: AGCAGTAGGGAATCTTCCA R: CACCGCTACACATGGAG	(Christensen et al., 2014)

### Gene Expression

Total RNA was isolated from approximately 100 mg of ileal, and cecal mucosa, which were homogenized in 0.2 mL of chloroform and 1 ml RNA Extracol (EURx, Gdansk, Poland) using a TissuesRuptor homogenizer (Qiagen GmbH, Hilden, Germany). RNA was purified from the solution and contaminant using a GeneMATRIX Universal RNA Purification Kit (EURx, Gdansk, Poland) following the manufacturer's instructions. Each RNA sample was quantitatively and qualitatively evaluated using the NanoDrop 2000 (Thermo Scientific Products). Gene expression analysis was performed for the gene panel, which included innate immune response genes (*IL-2, IL-4, IL-6, IL-8, IL-10, IL-12, IL-17, IL1-β, IFNγ, IFNβ)*, host defense peptides (*AvBD1, CATHL2)*, nutrient sensing genes (*FFAR2, FFAR4, GLUT1, GLUT2, GLUT5)* and barrier function genes (*MUC6, CLDN1, TJAP). ACTB* and *G6PDH* were used as reference genes (Table 3). Gene expression analysis was performed by qPCR with initial reverse transcription. cDNA was synthesized using the Maxima First Strand cDNA Synthesis Kit for RT-qPCR (Thermo Scientific/Fermentas, Vilnius, Lithuania). The qPCR reaction was performed using LightCycler 480 II. The qPCR reactions mixture contained Maxima SYBR Green qPCR Master Mix (Thermo Fisher Scientific, Waltham, MA), 1 μM of each primer specific to the target gene (synthesized by Sigma-Aldrich, Schnelldorf, Germany) and 70 ng of cDNA. The thermal profile of the qPCR reaction was carried out as follows: initial denaturation at 95°C for 15 min, followed by 40 cycles of denaturation at 95°C for 15 s, annealing at 58°C for 15 s, and elongation at 72°C for 45 s and melting curve. The ΔΔCT algorithm calculated relative gene expression. The amount of the target gene was calculated by the 2^-ΔΔCT^ formula (Livak and Schmittgen, 2001).

**Table 3:** Genes primer sequences used in RT-qPCR reaction (F- Forward primer; R-Reverse primer).

Gene	Name	Primer sequences (Forward/Revers)	Reference
*ACTB*	Actin, beta	F: CACAGATCATGTTTGAGACCTT R: CATCACAATACCAGTGGTACG	(Slawinska et al., 2019)
*G6PDH*	Glucose 6 phosphate dehydrogenase	F: CGGGAACCAAATGCACTTCGT R: GGCTGCCGTAGAGGTATGGGA	(Slawinska et al., 2019)
*IL1β*	Interleukin 1 beta	F: GGAGGTTTTTGAGCCCGTC R: TCGAAGATGTCGAAGGACTG	(Slawinska et al., 2019)
*IL-2*	Interleukin 2	F:GCTTATGGAGCATCTCTATCATCA R: TTGGGCAGGTTGAGGTTGTT	(Slawinska et al., 2019)
*IL-4*	Interleukin 4	F: GCTCTCAGTGCCGCTGATG R: GGAAACCTCTCCCTGGATGТС	(Biesek et al., 2021)
*IL-6*	Interleukin 6	F:AGGACGAGATGTGCAAGAAGTTC R: TTGGGCAGGTTGAGGTTGTT	(Sławinska et al., 2014)
*IL-8*	Interleukin 8	F:AAGGATGGAAGAGAGGTGTGCTT R: GCTGAGCCTTGGCCATAAGT	(Sławinska et al., 2014)
*IL-10*	Interleukin 10	F: CATGCTGCTGGGCCTGAA R: CGTCTCCTTGATCTGCTTGATG	(Biesek et al., 2021)
*IL-12*	Interleukin 12	F: TTGCCGAAGAGCACCAGCCG R: CGGTGTGCTCCAGGTCTTGGG	(Slawinska et al., 2019)
*IL-17*	Interleukin 17	F: CCGTCTTCTGCTGAGAGGAGTG R: ACCGTTGTTCCGTCCCATCAC	(Biesek et al., 2021)
*IFNβ*	Beta interferon	F: ACCAGATCCAGCATTACATCCA R: CGCGTGCCTTGGTTTACG	(Biesek et al., 2021)
*IFNγ*	Gamma interferon	F: ACACTGACAAGTCAAAGCCGC R: AGTCGTTCATCGGGAGCTTG	(Biesek et al., 2021)
*AvBD1*	Avian beta defensin 1	F: AAACCATTGTCAGCCCTGTG R: TTCCTTAGAGCCTGGGAGGAT	(Slawinska et al., 2019)
*CATHL2*	Cathelicidin 2	F: AGGAGAATGGGGTCATCAGG R: GGATCTTTCTCAGGAAGCGG	(Slawinska et al., 2019)
*CLDN1*	Claudin 1	F: TCTTCATCATTGCAGGTCTGTC R: AACGGGTGTGAAAGGGTCAT	(Slawinska et al., 2019)
*TJAP1*	Tight junction associated protein	F: AGGAAGCGATGAATCCCTGTT R: TCACTCAGATGCCAGATCCAA	(Slawinska et al., 2019)
*MUC6*	Mucin 6	F: TTCAACATTCAGTTCCGCCG R: TTGATGACACCGACACTCCT	(Slawinska et al., 2019)
*FFAR2*	Free fatty acid receptor 2	F: GCTCGACCCCTTCATCTTCT R: ACACATTGTGCCCCGAATTG	(Slawinska et al., 2019)
*FFAR4*	Free fatty acid receptor 4	F: AGTGTCACTGGTGAGGAGATT R: ACAGCAACAGCATAGGTCAC	(Slawinska et al., 2019)
*GLUT1*	Glucose transporter 1	F: AGATGACAGCTCGCCTGATG R: GTCTTCAATCACCTTCTGCGG	(Slawinska et al., 2019)
*GLUT2*	Glucose transporter 2	F: GGAGAAGCACCTCACAGGAA R: CAGGCTGTAACCGTACTGGA	(Slawinska et al., 2019)
*GLUT5*	Glucose transporter 5	F: ACGGTTCCCAGAGCAAGTTA R: GTCTTGCATGTATGGGGCTG	(Slawinska et al., 2019)

### Statistical Analysis

Statistical analysis was performed using SAS statistical software (SAS Enterprise Guide 8.3; SAS Institute Inc., Cary, NC). The significance of gene expression and the effect on bacterial abundance was analyzed by one-way ANOVA. However, the significance of the influence of intestinal section, substance and interaction (intestinal section x substance) was calculated using a 2-way ANOVA followed by Tukey's HSD post hoc test, for which the classifying variable (tissue and group) and the dependent variable (tested gene).

## RESULTS

### Production Data

Data are presented as hatchability rates in Table 1. The sum of fertilized and non-dead eggs, the number of hatched, crippled, weak chicks, and unhatched eggs was calculated. Values are given as sums and percentages of eggs laid and fertilized in Table 1.In the MOS3 group, a significantly lower number of eggs were placed in the hatcher on the 18th d of incubation (*P* = 0.034). During the egg candling, it was found that the developing chicken embryos were dead in 2 hatching eggs, which were visible through a bloody ring adhering to the eggshell of the eggs. After opening the eggs, it was found that death occurred on d 14 (medium mortality) and d 17 (late mortality) (Table 1).

There were no statistically significant differences in growth performance parameters (Table 4).

**Table 4:** Growth performance of chickens stimulated in ovo by different prebiotics.

Item^1^	Groups^2^					SEM	*P*-value
	Control	XOS3	XOS4	MOS3	MOS4		
BW (g)							
D 1	46.48	46.25	45.75	46.63	46.79	0.204	0.461
D 14	432.30	439.82	431.08	431.71	424.15	4.400	0.577
D 35	1962.72	1924.78	1926.04	1948.13	1877.15	16.550	0.892
D 42	2520.53	2480.86	2484.78	2546.49	2451.32	27.855	0.589
BWG (g)							
D 1 – 14	385.82	393.57	385.33	385.08	377.35	4.400	0.872
D 15 – 35	1530.42	1484.96	1494.96	1516.42	1453.00	14.566	0.880
D 36 – 42	557.80	556.08	558.74	598.35	574.17	24.928	0.539
Total	2474.05	2434.61	2439.03	2499.86	2404.53	27.857	0.986
FI (g)							
D 1 – 14	459.75	470.37	459.90	480.30	458.54	4.536	0.872
D 15 – 35	2498.13	2571.79	2352.10	2459.62	2372.71	36.077	0.525
D 36 – 42	1206.37	1340.89	1267.91	1256.83	1171.36	34.283	0.296
Total	4164.25	4383.04	4079.91	4196.75	4002.61	68.038	0.629
FCR (kg/kg)							
D 1 – 14	1.20	1.20	1.20	1.25	1.22	0.017	0.509
D 15 – 35	1.63	1.73	1.57	1.62	1.63	0.025	0.860
D 36 – 42	2.37	2.54	2.33	2.12	2.04	0.125	0.386
Total	1.69	1.80	1.68	1.68	1.67	0.022	0.771
Viability (%)	87.50	89.58	95.83	95.83	97.92	2.059	0.300

1 BW – body weight; BWG – body weight gain; FI – feed intake; FCR – feed conversion ratio; SEM, standard error of the mean

2 XOS3 – xylotriose, XOS4 – xylotetrose, MOS3 – mannotriose, MOS4 – mannotetrose

### Intestinal Microbiota Activity

In ovo stimulation with oligosaccharides did not affect ileal and cecal digesta pH. SCFA concentration in the ileum and ceca were similar in all groups and did not differ from the control group (Table 5). Also, there was no effect on ammonia concentration in the ceca of broiler chickens. Bacterial β-glucuronidase activity was considerably higher in ceca than in the ileum but was unaffected by in ovo stimulation with oligosaccharides (Table 6).

**Table 5:** Digesta pH and SCFA concentrations in the ileum and cecum of broiler chickens stimulated in ovo by different prebiotics.

Groups	Short chain fatty acids, μmol/g digesta						pH
	Acetate	propionate	isobutyrate	butyrate	isovalerate	valerate	
Ileum							
Control	12.44	1.58	0.27	1.62	0.07	0.24	6.41
XOS3	9.52	0.44	0.24	0.08	0.00	0.18	6.51
XOS4	9.88	0.55	0.41	0.00	0.03	0.25	6.61
MOS3	9.40	0.59	0.39	0.02	0.00	0.29	7.45
MOS4	7.90	0.56	0.32	0.27	0.00	0.22	6.82
SEM	0.878	0.178	0.031	0.228	0.010	0.022	0.140
P	0.6108	0.2263	0.3753	0.1112	0.1826	0.6092	0.1614
Cecum							
Control	59.41	18.49	1.30	9.09	1.06	1.11	7.41
XOS3	57.97	17.78	1.36	9.23	1.11	1.12	7.41
XOS4	44.87	14.40	1.14	6.93	1.01	0.96	7.72
MOS3	59.91	16.61	1.32	7.11	1.13	1.02	7.51
MOS4	57.33	20.33	1.29	8.35	1.12	1.13	7.33
SEM	1.968	0.852	0.047	0.411	1.086	0.039	0.062
P	0.0757	0.2382	0.6376	0.2474	0.9163	0.5478	0.3285

SEM, standard error of the mean, XOS3 – xylotriose, XOS4 – xylotetrose, MOS3 – mannotriose, MOS4 – mannotetrose.

**Table 6:** Ammonia concentration (µM/g digesta) and β-glucuronidase (U/g digesta) activity in the intestinal content of broiler chickens stimulated in ovo by prebiotics.

Groups	Ammonia	β-glucuronidase	
	Cecum	Ileum	Cecum
Control	38.24	0.09	141.38
XOS3	30.95	0.41	140.52
XOS4	29.02	0.31	149.62
MOS3	35.75	0.19	194.65
MOS4	43.78	1.37	149.35
SEM	1.801	0.207	14.53
P	0.0757	0.2926	0.7766

SEM, standard error of the mean, XOS3 – xylotriose, XOS4 – xylotetrose, MOS3 – mannotriose, MOS4 – mannotetrose

### Gene Expression

**Figure 1:**
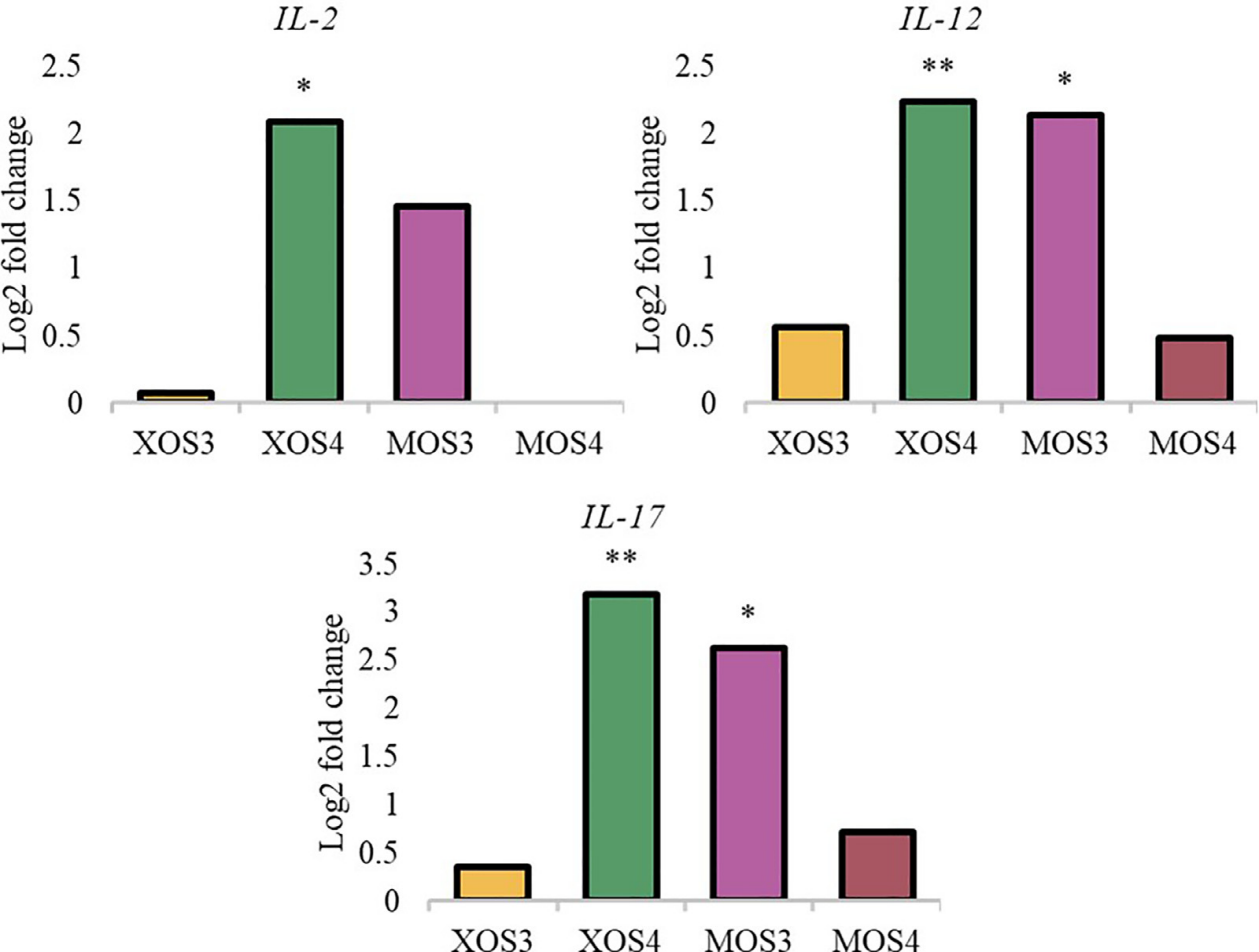
Expression of innate immune response genes in the caecum. The relative gene expression was calculated by the ΔΔCT algorithm and the amount of the target gene was calculated by the 2-ΔΔCT formula. Asterisks indicate means that differ significantly from the control group at *P*≤ 0.05 (*), *P*≤ 0.01 (**), or *P*≤ 0.001 (***). XOS3 – xylotriose, XOS4 – xylotetrose, MOS3 – mannotriose, MOS4 – mannotetrose.

**Figure 2:**
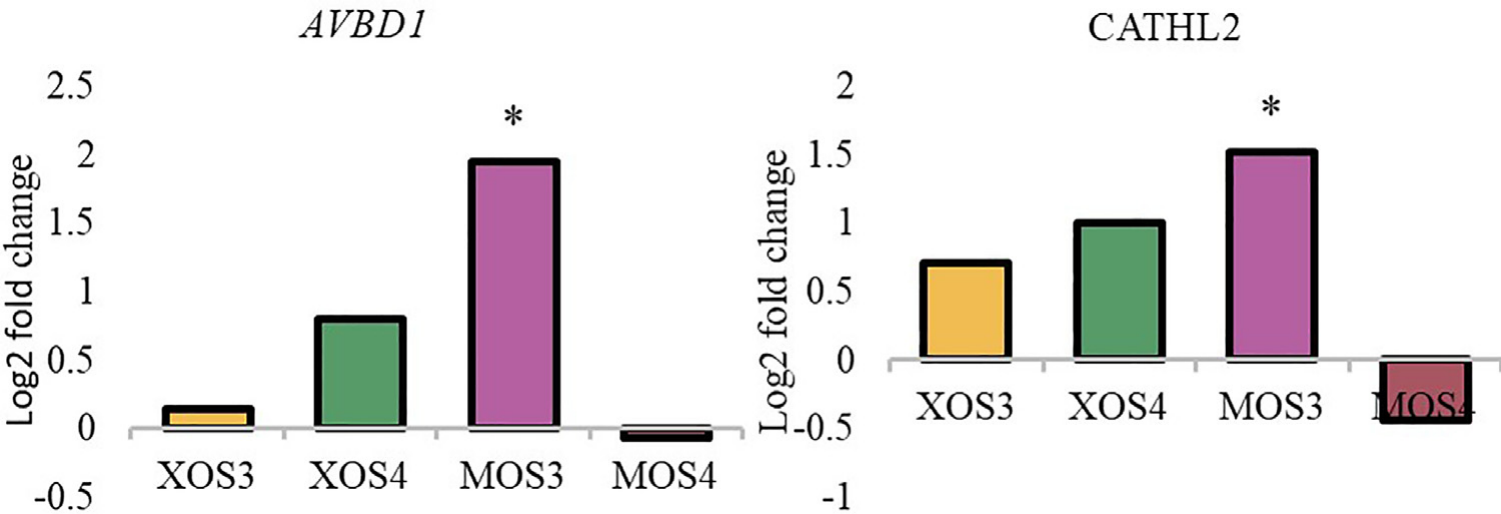
Expression of host defense peptides genes in the caecum. The relative gene expression was calculated by the ^ΔΔ^C_T_ algorithm and the amount of the target gene was calculated by the 2^-ΔΔCT^ formula. Asterisks indicate means that differ significantly from the control group at *P*≤ 0.05 (*), *P*≤ 0.01 (**), or *P*≤ 0.001 (***). XOS3 – xylotriose, XOS4 – xylotetrose, MOS3 – mannotriose, MOS4 – mannotetrose.

**Figure 3:**
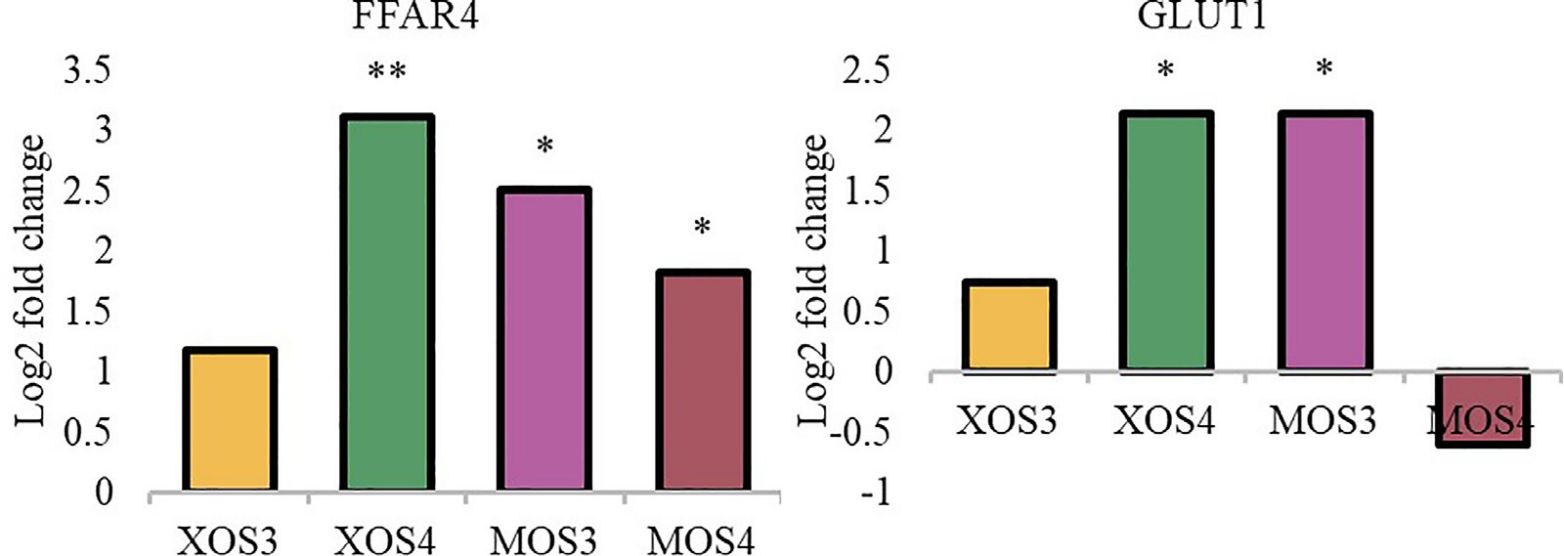
Expression of nutrient sensing genes in the caecum. The relative gene expression was calculated by the ΔΔCT algorithm and the amount of the target gene was calculated by the 2-ΔΔCT formula. Asterisks indicate means that differ significantly from the control group at *P*≤ 0.05 (*), *P*≤ 0.01 (**), or *P*≤ 0.001 (***). XOS3 – xylotriose, XOS4 – xylotetrose, MOS3 – mannotriose, MOS4 – mannotetrose.

Table 7 shows the significance of the effects of gut section (ileum, cecum) substance (XOS3, XOS4, MOS3, MOS4) and interaction (gut section x substance). In the ileum, no significant differences were noted in the level of gene expression after in ovo stimulation with prebiotics. Administration of prebiotics in ovo significantly affected gene expression in the cecum. Stimulation caused significant changes in the expression of innate immune response genes, host defense peptides, and nutrient-sensing genes. The MOS3 group shows an increase in the expression of all tested genes. The XOS4 group shows an increase in the expression of interleukins and nutrient-sensing genes. Figures 1, 2, and 3 show statistically significant changes in gene expression levels in the cecum.

**Table 7:** Effects of experimental groups, intestinal segment and their interaction on genes expression in chicken intestinal mucosa.

Gene	Intestine^1^	Substance^2^	Intestine x Substance^3^
Cytokine genes			
*IL2*	< 0.0001	< 0.05	Ns
*IL12*	< 0.0001	ns	Ns
*IL17*	ns	ns	< 0.01
**Host defence peptide (HDP) genes**			
*AVBD1*	< 0.01	ns	< 0.05
*CATHL2*	< 0.0001	ns	Ns
**Nutrient sensing genes**			
*FFAR4*	< 0.001	ns	< 0.0001
*GLUT1*	< 0.001	ns	< 0.05

Effects.

1 Intestinal segment (ileum, cecum).

2 In ovo delivery of XOS3, XOS4, MOS3, MOS4.

3 The interaction between intestinal segment and in ovo delivery substances.

### Relative Abundance of Bacteria

**Figure 4:**
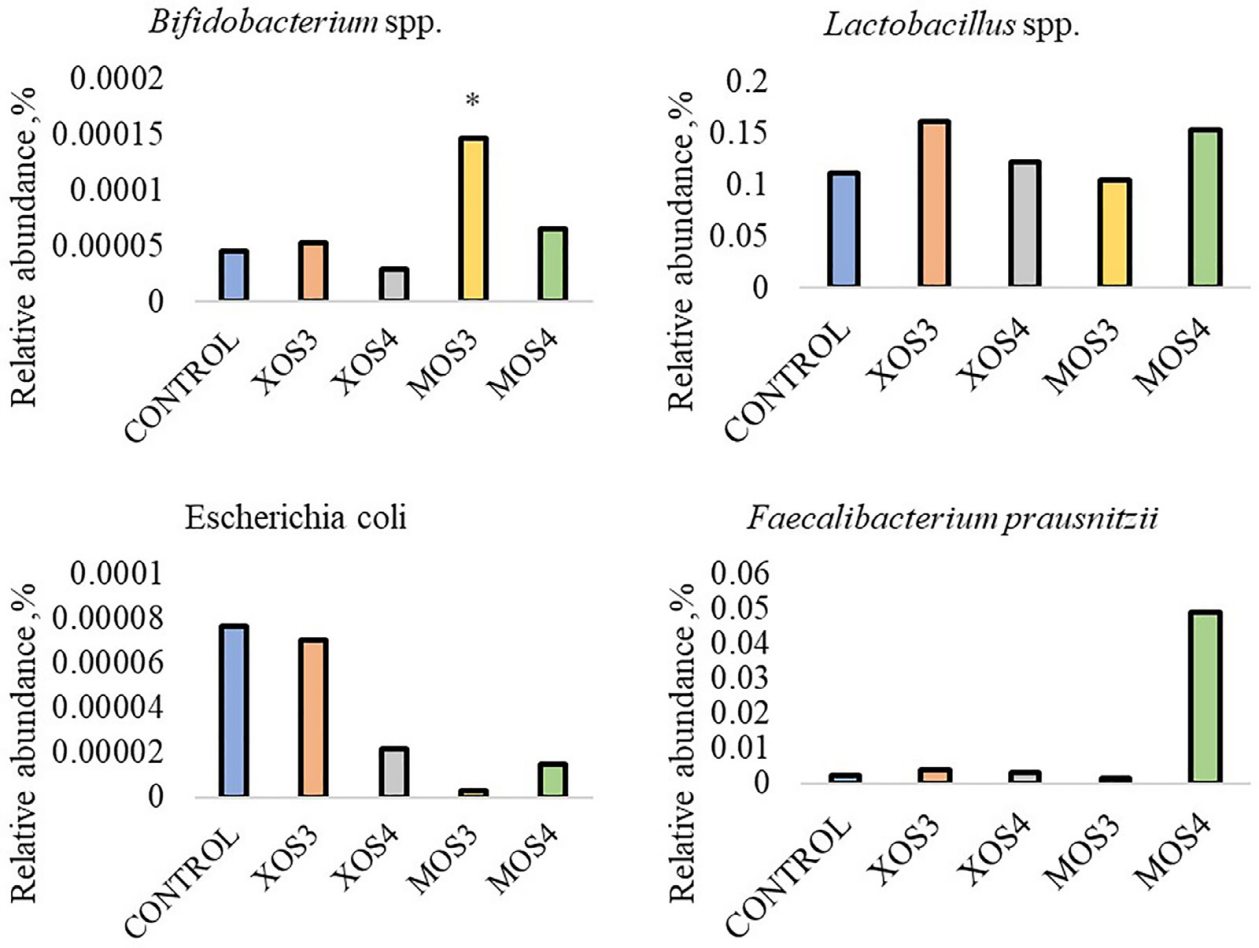
Relative abundance of bacteria in the ileum contents after in ovo stimulation with prebiotics. Asterisks indicate means that differ significantly from the control group at P ≤ 0.05 (*), P ≤ 0.01 (**), or P ≤ 0.001 (***). XOS3 – xylotriose, XOS4 – xylotetrose, MOS3 – mannotriose, MOS4 – mannotetrose.

**Figure 5:**
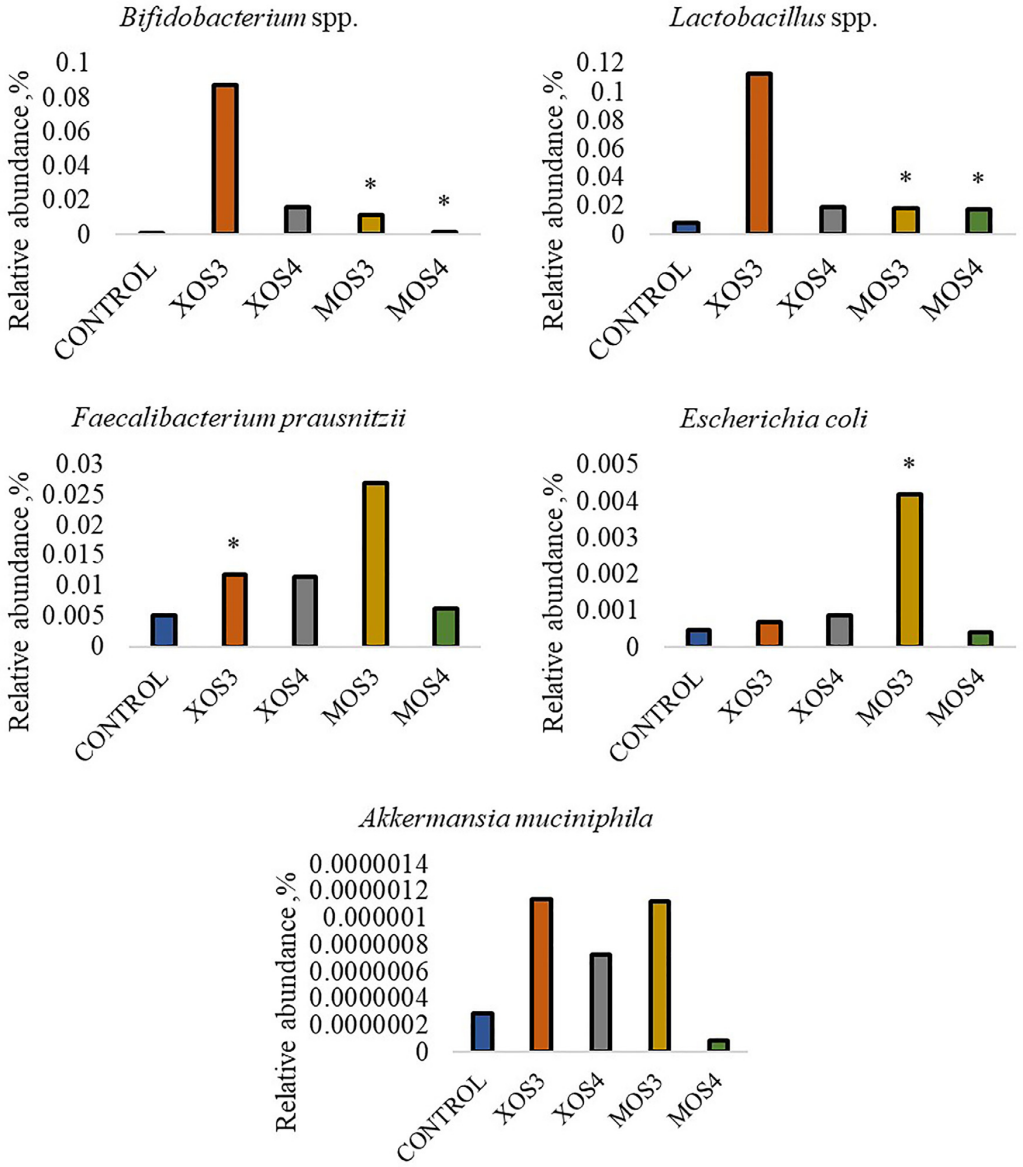
Relative abundance of bacteria in the cecal contents after in ovo stimulation with prebiotics. Asterisks indicate means that differ significantly from the control group at *P* ≤ 0.05 (*), *P* ≤ 0.01 (**), or *P* ≤ 0.001 (***). XOS3 – xylotriose, XOS4 – xylotetrose, MOS3 – mannotriose, MOS4 – mannotetrose.

Prebiotic administration in ovo had a significant effect on the relative abundance of bacteria in the ileum and cecum. In the case of the abundance of *Bifidobacterium* spp., their amount in the ileum in the MOS3 group increased more than 4 times compared to the remaining groups (*P* < 0.05). In the cecum, in the MOS3 group, there was a more than 2 times increase in the abundance of *Bifidobacterium* spp., *Lactobacillus* spp., and *Escherichia coli* as compared to the control group (*P* < 0.05). The relative abundance of *Lactobacillus* spp. was more significant in the cecum of chickens in the MOS4 group than in other groups (*P* < 0.05). Figure 4 shows the relative abundance of bacteria in the ileum, while Figure 5 shows the relative abundance of bacteria in the cecum.

## DISCUSSION

The current study is a continuation of the research on the impact of in ovo stimulation with bioactive substances on chicken intestinal microbiota. Particularly important, in this case, are direct and indirect effects of prebiotics on intestinal health in broiler chickens. Previously, the positive effect of galacto-oligosaccharides on the development of the microbial population and the gene expression in the mucosa in each section of the intestine was described by Slawinska et al., 2019 (Rinttilä and Apajalahti, 2013). Modification of the intestinal microbiota is possible already at the stage of embryo development by administration of bioactive substances directly to the egg. In ovo stimulation is a process involving the injection of specific bioactive substances, including prebiotics, into the egg's air chamber on d 12 of incubation (Siwek et al., 2018). XOS and MOS used in the research belong to oligosaccharides of hemicellulose origin. They are obtained, among others, from guar gum, corn cobs, palm kernel expeller, and locust bean gum. The basis for in ovo stimulation with prebiotic MOS is its interaction with the host organism's cells to induce an immune response, the effect on the activity of enzymes, and the modulation of the microbiota. Mannan-oligosaccharide is produced by beta-mannases derived from bacteria and fungi MOS. It increases animal performance by reducing harmful bacteria while stimulating an increase in the abundance of beneficial bacteria (Rezaei et al., 2015; Ayimbila and Keawsompong, 2022). Manno-oligosaccharides stimulate nutrient absorption and immune response. Additionally, they increase the production of SCFAs, including acetate, butyrate, and propionate. The present study showed an up-regulation of *IL-2, IL-12,* and *IL-17* gene expression in the cecum in of birds stimulated with XOS3 and MOS4. According to the results obtained by Kim et al., 2014, an increase in the expression of *IL-12* and *IL-17* may indicate the presence of infection in chickens or the formation of inflammation. This is an innovative finding because, according to the literature, both prebiotics are used to activate the intestinal microbiota by increasing the abundance of beneficial microbes (Peng et al., 2020). In the studies described by Ayimbila and Keawsompong, 2022, it was proven that MOS stimulates the growth of *Lactobacillus* spp. and *Bifidobacteria* spp. in the intestines of broilers. These results are consistent with the obtained values, which show more than a 2-fold increase in *Lactobacillus* spp. and *Bifidobacterium* spp. in the cecum and a several-fold increase in *Bifidobacterium* spp. in the ileum. The results proved the prebiotic effect of MOS, which modulates the environmental conditions in the digestive tract of chickens and ensures the appropriate microbiocenosis by stimulating the growth of beneficial bacteria. In groups subjected to in ovo stimulation with MOS, an increase in the expression of host defense peptides *AvBD1, CATHL2* was observed in the cecum. These genes correspond to the innate immunity and the mucosal defense. Intestinal HDP genes are associated with mucins, creating an immunological and mechanical barrier of the host. Their main functions include antimicrobial activity. They also participate in the process of wound healing as well as immunomodulation and chemotaxis. (Akbari et al., 2008; Slawinska et al., 2019) HDP gene expression depends on microbial modulation. Defensins, which include *AvBD1*, are defence peptides expressed mainly in epithelial cells. Their main function is to protect the host against bacterial, viral, and fungal infections. They have the ability to inhibit the growth of pathogenic bacteria. In turn, cathelicidins, including that encoded by *CATHL2* gene, are host-protective proteins that play a significant role in the innate and adaptive immunity. Similarly to defensins, they can eliminate pathogens and modulate the immune response (Dunislawska et al., 2022). Higher expression of *AvBD1* and *CATHL2* genes in the current study may indicate an inflammatory response. This might suggest a disease state of the host organism, and necessity to defend the body against pathogens. Increased expression of both genes in the MOS3 group might be related to a significant increase in the abundance of *Escherichia coli*.

XOS has a bifidogenic effect, improves the absorption of nutrients, and stimulates the immune response. Dietary supplementation with XOS can improve growth performance of chickens by positively enhancing immune function and improving gut health. As a prebiotic, XOS promotes the growth of beneficial intestinal bacteria and increases the production of SCFA in the intestines of broilers (Wang et al., 2021). In the XOS-stimulated groups, the abundance of *Lactobacillus* bacteria increased. SCFA are the main energy source for the intestinal microbiota and intestinal epithelial cells. Ding et al., 2018 observed that XOS improved gut health and immune function by increasing SCFA, including butyric acid and *Bifidobacteria* counts in the cecum of chickens. The results obtained from the current research confirm the stimulatory effect of XOS3 and XOS4 on *Bifidobacterium* spp. population in the cecum, and of MOS3 and MOS4 in both intestinal sections. The current research demonstrated an increased expression of *FFAR4* and *GLUT1* genes in the cecum after stimulation with XOS4 and MOS3. Both genes are nutrient-sensing genes. Groups that showed a significant increase in *GLUT1* expression appear advantageous due to its functions. *GLUT1* is responsible for facilitating basal glucose uptake, essential for most cells' growth and development (Kono et al., 2005).

The intestinal microbiota produces many metabolites which may affect the host. SCFA are the end-products of carbohydrate and protein fermentation, while branched-chain fatty acids (iso-butyrate, iso-valerate), ammonia, amines, as well as phenolic and indolic compounds are formed during proteolysis (Taciak et al., 2017). In ovo stimulation of broiler chickens with oligosaccharides did not affect the indices of microbial activity in the ileum and cecum. Only a tendency toward a reduction of acetic acid concentration was found in the cecum of birds stimulated with XOS4. This trend may suggest that the population of bacteria being its producers, for example, *Bacteroides-Prevotella* group (Louis et al., 2007) was reduced. However, in the current study, their abundance was not studied. Therefore, more detailed analysis of microbiota composition should be done in the future research to determine the effect of in ovo stimulation with prebiotics on microbial ecology of broiler chickens. Since acetic acid is a bacterial metabolite, which can be utilized in lipogenesis in the liver and as a fuel for skeletal muscles (2002) the effect of prebiotic administration during embryonic development on peripheral tissues of chickens should be also analysed.

The current research showed also a tendency toward higher ammonia concentration in the cecum of birds stimulated in ovo with MOS4. The results may suggest that this oligosaccharide has a potential to intensify deamination of amino acids or degradation of blood urea as these reactions lead to ammonia release (Windey et al., 2012). The concentration of ammonia in the intestinal content also depends on the absorption by the epithelium (Lupton and Marchant, 1989; Windey et al., 2012). Thus, the tendency to its higher level found in the MOS4 group might result from the intensified proteolysis or impaired absorption. It may be also speculated that MOS4 inhibited assimilation of ammonia by the cecal bacteria, which use it for the synthesis of their own protein (Blachier et al., 2007).

Beside SCFA and ammonia, bacterial β-glucuronidase activity was also measured in the current study. This enzyme hydrolyses glucuronides synthesized in the liver and secreted with the bile (Pellock and Redinbo, 2017). Thus, it participates in entero-hepatic circulation of substances formerly detoxified in the liver. The activity of this enzyme was considerably greater in the cecal than ileal digesta, which is line with the results of analysis of the relative abundance of bacteria. The β-glucuronidase activity was found in *Bacteroides, Bifidobacterium, Eubacterium*, and *Ruminococcus*. Its gene was described for *E. coli, Lactobacillus gasseri*, and *Staphylococcus* spp., and identified also in *Clostridium perfringens* (AKAO, 1999, 2000; Russell and Klaenhammer, 2001; Beaud et al., 2005). In the current study, the abundance of *E. coli* and *Bifidobacterium* spp. was much greater in the cecum than in the ileum, which may partially explain the difference in β-glucuronidase activity between these segments. In the current study, it was demonstrated that in ovo administration of oligosaccharides did not affect the activity of this enzyme despite the abundance of *E.coli* and *Bifidobacterium* spp. differed between groups. However, these bacteria are not predominating in the intestinal content of broiler chickens and changes in their population size seems to have no impact on β-glucuronidase activity. The lack of effect of prebiotics administered in ovo indicate that the bile secretion from the liver was not affected in chickens. The fact that all birds were fed the same diets was also of importance. Each diet for chickens (starter, grower, and finisher) was based on cereals and soybean meal, the latter being a source of isoflavones (Tuśnio et al., 2014). These compounds are metabolized in the liver by binding to glucuronic acid and then secreted as glucuronides with bile (Dabek et al., 2008). Tuśnio et al. 2020 demonstrated that feeding diets without soybean meal reduced the activity of β-glucuronidase in the colon of pigs. In the current research, the soybean meal content in a diet was the same for each group, which ensured similar availability of substrates (isoflavone glucuronides) for the intestinal microbiota.

## CONCLUSIONS

1. In ovo stimulation with prebiotics resulted in significant changes in the genes expression, such as those involved in the innate immune response, host defense peptides and nutrient sensing genes. These changes imply a potential improvement in the overall immune capacity and metabolic regulation of the developing chicken embryo. Potentially leading to improved broiler health and growth performance after hatching.
2. In ovo prebiotic injection stimulates the growth of beneficial bacteria in chickens intestines. Such early stimulation of the microbiota can lead to better digestion, nutrient absorption and a more efficient immune system in chickens.

## DISCLOSURES

The authors declare no conflicts of interest.
